# The Roles of TRAF3 in Immune Responses

**DOI:** 10.1155/2023/7787803

**Published:** 2023-02-16

**Authors:** Mengjiao Lin, Xiaoli Ji, Yan Lv, Dawei Cui, Jue Xie

**Affiliations:** ^1^Department of Blood Transfusion, The First Affiliated Hospital, Zhejiang University School of Medicine, Hangzhou 310003, China; ^2^Department of Ultrasound, Wenzhou City People's Hospital, Wenzhou 325100, China

## Abstract

Seven tumor necrosis factor receptor- (TNFR-) associated factors (TRAFs) have been found in mammals, which are primarily involved in the signal translation of the TNFR superfamily, the Toll-like receptor (TLR) family, and the retinoic acid-inducible gene I- (RIG-I-) like receptor (RLR) family. TRAF3 is one of the most diverse members of the TRAF family. It can positively regulate type I interferon production while negatively regulating signaling pathways of classical nuclear factor-*κ*B, nonclassical nuclear factor-*κ*B, and mitogen-activated protein kinase (MAPK). This review summarizes the roles of TRAF3 signaling and the related immune receptors (e.g., TLRs) in several preclinical and clinical diseases and focuses on the roles of TRAF3 in immune responses, the regulatory mechanisms, and its role in disease.

## 1. Introduction

**Figure 1:**
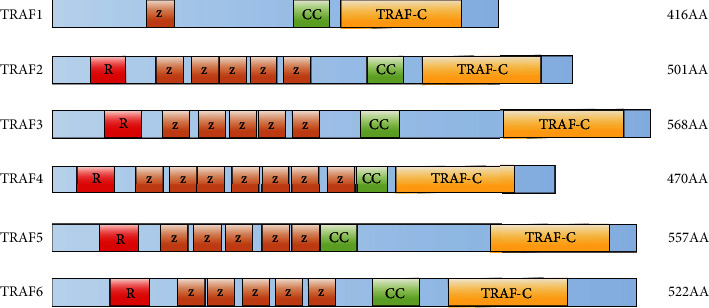
The six human TNFR-associated factor (TRAF) proteins that contain a C-terminal TRAF domain are shown. All TRAFs (except for TRAF1) contain an N-terminal RING finger domain (a signature motif of E3 RING finger ubiquitin ligase; labeled R) and several zinc finger motifs (labeled Z). The TRAF domain contains a coiled-coil region (labeled CC) and a C-terminal TRAF-C domain (also known as a meprin and TRAF homology (MATH) domain). AA: amino acids.

The TRAF family has seven members, corresponding to TRAF1-TRAF7, with a similar common structure. Except for TRAF1, all members contain the Really Interesting New Gene (RING) finger domain at their N-terminal, which gives TRAFs E3 ubiquitin ligase activity. RING finger-mediated protein ubiquitination is primarily responsible for catalyzing substrate ubiquitination and activating the downstream signaling pathway. Except for TRAF7, all TRAFs have a common characteristic TRAF domain at the C-terminal that is divided into TRAF-N and TRAF-C (Figure 1). TRAF-N is responsible for mediating TRAF homotrimerization. At the same time, TRAF-C is responsible for binding to upstream regulators like TNFR2, CD40, and the BAFF receptor (BAFFR) or intermediate adaptor proteins such as TNFR1-associated death domain protein (TRADD) and IL-1R-associated kinase (IRAK) family. TRAF-C and the TRAF-N can both bind to downstream effector proteins such as a cellular inhibitor of apoptosis (cIAP) and NF-*κ*B-inducing kinase (NIK), which can bind to TRAF-N of TRAF2 and the TRAF-C of TRAF3, respectively [1–3].

TRAF3 is one of the most multifunctional TRAF molecules. The human TRAF3 gene is found on chromosome 14, and its cDNA is approximately 1.7 kb long. The encoded protein has 568 amino acids, and its molecular weight is 64 kD. TRAF3 is a typical TRAF family member, with a RING finger motif at the N-terminus, a zinc finger structure in the middle, and a TRAF domain at the C-terminus. TRAF3 forms a mushroom-like homotrimer complex through the *α*-helix structure at the end of TRAF-N and the antiparallel *β*-sheet structure at the end of TRAF-C. This trimer chemical structure provides an important foundation for signal transmission from the extracellular to the intracellular receptor. The antiparallel *β*-sheet structure has a hydrophobic gap that can bind to small peptides containing at least 20 amino acids. The CD40, BAFFR, and lymphotoxin *β* receptor (LT*β*R) in the TNFR family can interact with the hydrophobic gap of TRAF3. The binding parts of these proteins have similar sequences, collectively known as TRAF interaction motifs (TIM). This major TRAF-binding motif has the consensus sequence (P/S/A/T)X(Q/E)E, which can be identified in most of the known TRAF-binding sequences from TNFRs. The minimal consensus motif in TRAF-binding proteins, including TNFR family members, for TRAF3 interaction is Px(Q or E)E# (x: any amino acid, #: acidic or polar amino acids are favored) [4, 5]. TRAF3 is widely expressed in almost all tissues, including the brain, heart, spleen, lung, liver, and lymph. The widespread expression of TRAF3 also indicates that it plays a significant role in various physiological processes [6].

The TRAF family was involved in binding to the TNFR family and mediating downstream signaling activation. TNFR family members lack a motif that can recruit downstream tyrosine kinases, requiring intermediate proteins, such as TRAFs, to mediate signal transmission downstream. As important adaptor proteins, TRAFs initiate signal transduction by interacting with receptors and mediating substrate ubiquitination. TRAF1/2/5/6 share the same biological function as they can promote the activation of classical NF-*κ*B signaling and induce the activation of MAPK signaling [7–9]. However, TRAF3 is unique in the TRAF family. It does not stimulate the classical NF-*κ*B and MAPK signaling pathways. Therefore, it received less attention in early studies than TRAF2 and TRAF6 [10, 11]. Generally, TRAF2, 5, and 6 are activators or/and enhancers of the classical NF-*κ*B signaling pathway, while TRAF3 acts mainly as an inhibitor of the nonclassical NF-*κ*B pathway through NIK degradation. In addition, TRAF3 knockout mice die young, which hinders further research on TRAF3 function [12]. TRAF3 is broadly involved in different receptor-mediated signaling pathways. In recent years, there have been more and more studies on TRAF3 in diseases. Here, we reviewed these new findings about how TRAF3 mediates different signaling cascades and modification and activation of TRAF3. This review will help fully understand the immune function and molecular mechanism of TRAF3 in immune diseases and provide a theoretical basis for disease treatment.

## 2. TRAF3 Plays Different Functions in Different Signaling Pathways

**Figure 2:**
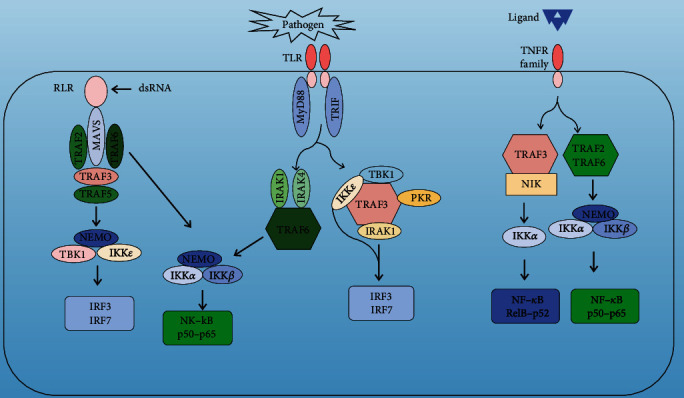
Engagement of Toll-like receptor (TLR) and retinoic acid-induced gene-1 like receptor (RLR) triggers two main signaling pathways that are dependent on either myeloid differentiation primary response protein 88 (MYD88) or TIR domain-containing adaptor protein inducing IFN*β* (TRIF). TNFR-associated factor 3 (TRAF3) is recruited to both the MYD88-assembled and TRIF-assembled signaling complexes which upregulate IRF3 and IRF7 activation, depending on the catalytic activity of TANK-binding kinase1 (TBK1) and the I*κ*B kinase*ε* (IKK*ε*). TRAF6 is essential for activating most known MYD88-dependent effector pathways, including the nuclear factor-*κ*B (NF-*κ*B). The classical NF-*κ*B pathway, which is triggered by RLRs, Toll-like receptors (TLRs), and TNF receptors (TNFRs), depends on the catalytic activity of the I*κ*B kinase (IKK) catalytic subunit IKK*α* and IKK*β*. The nonclassical NF-*κ*B pathway is mainly activated by a subset of TNFR and depends on the catalytic activity of IKK*α*, which is activated by NF-*κ*B-inducing kinase (NIK), the turnover of which is regulated by TRAF3. Activated IKK*α* phosphorylates p100, freeing its N-terminal portion (p52), which enters the nucleus together with RELB.

TRAF3 plays different roles in the three signaling pathways mediated by TNFR, TLR, and RLR by participating in different protein complexes. It can positively regulate the type I interferon production while negatively regulating the activation of MAPK, classical, and nonclassical NF-*κ*B signals (Figure 2). TLR and RLR are two types of pattern recognition receptors (PRR) that induce the expression of type I interferon and proinflammatory cytokines by recognizing pathogen-associated molecular patterns (PAMPs) and activating signal cascade reactions to kill pathogenic microbes [13–15]. The TNFR family contains 29 members, including LT*β*R, CD40, and BAFFR, which are expressed in different cells and perform various functions [16]. They significantly affect immune responses, organ growth, development, and intracellular homeostasis maintenance. TRAF3 is one of the most diverse members of the TRAF family, with roles in various signaling pathways. The effect of its structure on function needs to be explored more precisely [17].

### 2.1. TRAF3 Promotes the Production of Type I Interferon through TLR and RLR

TRAF3 is very important for type I interferon production through TLR and RLR. TRAF3 knockout in macrophages and dendritic cells demonstrates a significant decrease in type I interferon [18, 19]. TLR is located on the cell and endosome membranes that stimulate the production of type I interferon and inflammatory factors by recognizing different PAMPs. Through the TIR domain, TLR recruits downstream connectors such as myeloid differentiation primary response gene 88 (MyD88) and TIR domain-containing adaptor protein inducing IFN*β* (TRIF) [20]. Except for TLR3, all TLR mediates MyD88-dependent signaling pathways. TLR3 and endocytosed TLR4 receptors can form TRIF-dependent signaling pathways. TLR3 primarily recognizes double-stranded RNA (dsRNA), while TLR4 recognizes bacterial lipopolysaccharides (LPS). Following external stimulation, TLR3/4 recruits a common adaptor protein TRIF [21]. TRAF3/6 binds to the N-terminal of TRIF. TRAF3 promoted the ubiquitination of K63, providing a platform for NF-*κ*B essential modulator (NEMO) and TANK-binding kinase/the I*κ*B kinase*ε* (TBK1/IKK*ε*) kinase complex [22]. Furthermore, TRAF3 also promotes the phosphorylation of IRF3 and the production of type I interferon [23–25]. TRAF6 activates the NF-*κ*B pathway by ubiquitinating itself and NEMO [26–30].

RLR includes retinoic acid-inducible gene I (RIG-I), melanoma differentiation-associated protein 5 (MDA5), and laboratory of genetics and physiology 2 (LGP2), which primarily recognizes dsRNA in the cytoplasm. After RLR recognizes the RNA virus in the cytoplasm, its conformation changes to the CARD domain, which binds to the CARD domain of the splicing protein mitochondrial antiviral signaling protein (MAVS) and promotes oligomerization of MAVS. Oligomeric MAVS binds to TRAF3, which is then ubiquitinated and activated by K63, and then recruits NEMO and TBK1/IKK*ε* complex to activate IRF3 and initiates interferon expression [31–34].

MAVS can be linked with TRAF2/3/5/6 through N- and C-terminal TIM sequences. Through the TRAF family, RIG signals can be divided into two distinct pathways: MAVS binds to TRAF2/6 to activate the I*κ*B-*α*/*β* kinase (IKK *α*/*β*) and NF-*κ*B signaling pathway, and MAVS binds to TRAF3 to start the production of type I interferon [35]. The TIM motif 455-PEENEY-460 at the C-terminal of MAVS can be combined with TRAF3. TRAF3 will not bind to MAVS, nor can it induce the interferon expression after mutation in TIM sequences.

However, the N-terminal TIM sequences did not affect TRAF3 binding and interferon expression. Concurrently, TRAF3 Y440A and Q442A are two important amino acid sites. After their mutations, TRAF3 will not bind to MAVS and induce the interferon expression [25, 36, 37].

### 2.2. TRAF3 Negatively Regulates the Production of Classical NF-*κ*B Signals and Inflammatory Cytokines through TLR

TLR-mediated MyD88-dependent signals activate NF-*κ*B signals through TRAF6, promoting inflammatory cytokine production [38, 39]. However, studies revealed that TRAF3 binds to both MyD88 and TRIF. TLR4 can mediate both MyD88 and TRIF signaling pathways. TRAF3 can be degraded in the MyD88-dependent pathway when TLR4 is activated. The complexes recruited by MyD88 include TRAF6, TRAF3, NEMO, and cIAP [40, 41]. TRAF6 can mediate the K63 ubiquitination of cIAP and activate it to function as an E3 ubiquitin ligase. Activated cIAP can mediate the K48 ubiquitination of TRAF3, which leads to TRAF3 degradation into the proteasome. Therefore, it stimulates the secretion of inflammatory factors in the TLR4 signaling pathway while inhibiting the production of type I interferon. And cIAPs were not found in the complex recruited by TRIF but were detected in the complex of MyD88. TRAF3 degradation will be the first step in activating the MyD88-dependent signaling pathway due to its inhibitory effect on this signaling pathway, but the mechanism of inhibition remains unknown. Therefore, TRAF3 is critical in coordinating the balance between type I interferon and inflammatory factors [42–44].

### 2.3. TRAF3 Negatively Regulates MAPK Signals through TNFR and TLR4

TRAF3 regulates MAPK signals through TNFR and TLR4. MAPK signaling is important in many physiological activities, including inflammation, apoptosis, invasion, and metastasis of tumor cells. TRAF3 was discovered to bind to CD40; however, unlike TRAF2/5/6, overexpression of TRAF3 suppresses CD40-mediated MAPK signaling. This important function of TRAF3 was identified by studying CD40 and TLR4. When CD40 is activated, many proteins are recruited to the cytoplasmic region around the receptor. These proteins are divided into two complexes primarily composed of MEK kinase-1 (MEKK1) or TGF *β*-activated kinase 1 (TAK1). TRAF3, cIAP, UBC13, and NEMO are all present in both complexes. Moreover, the MEKK1 complex includes TRAF2 and MEKK1, and the TAK1 complex includes TRAF6 and TAK1. TLR4 recruits the TAK1 complex through MyD88. These two complexes eventually phosphorylate MEKK1 and TAK1, essential for activating the MAPK signaling pathway. The complex proteins regulate the activation and inhibition of the MAPK signaling pathway. First, TRAF3 inhibits the release of MEKK1 and TAK1 into the cytoplasm; second, cIAP mediates the degradation of TRAF3 to remove its inhibitory function. TRAF2 and TRAF6 are activated by self-K63 ubiquitin activation after receptor stimulation. The formation of a ubiquitin chain strengthens the complex and recruits NEMO. TRAF2 and TRAF6 stimulation activates cIAP, which mediates the K48 ubiquitin degradation of TARF3. According to this model, knocking out of cIAP or its function inhibition or overexpression of TRAF3 can prevent the activation of MAPK signals. However, the mechanism by which TRAF3 inhibits TAK1 and MEKK1 remains unknown [42, 45, 46].

### 2.4. TRAF3 and Nonclassical NF-*κ*B Signal

Members of nonclassical NF-*κ*B mediated by the TNFR family include CD40, BAFFR, LTbR, RANK, TNFR2, TWEAK, and CD27. The p52/RelB heterodimer mediates the nonclassical NF-*κ*B pathway, with NIK as a key kinase. Unstimulated cells have stable low levels of NIK, and TRAF3 protein can interact with NIK, triggering continuous ubiquitin-proteasome degradation of NIK protein. TRAF3 was degraded, and NIK accumulated when the cell surface receptors LT*β*R, CD40, and BAFFR were activated. After NIK phosphorylate IKK*α*, the E3 ubiquitin ligase SCFpTrCP mediates ubiquitin modification of p100, the precursor of NF-*κ*B, and p100 is then processed into mature p52. p52 and Rel-B form an active heterodimer in the nucleus, which induces corresponding gene expression [47, 48].

The nonclassical NF-*κ*B pathway is critical for secondary lymphoid tissue development and adaptive immune response. TRAF3 knockdown causes NIK accumulation in cells and overactivation of the nonclassical NF-*κ*B pathway, resulting in the early death of mice. The mice survived when the NF-*κ*B2-p100 and TRAF3 genes were both knocked out. In addition, the studies revealed that LT*β*R, CD40, and BAFFR gene knockdown mice have similar phenotypes. This indicates that the nonclassical NF-*κ*B signaling pathway is inhibited. These studies further demonstrated that the TRAF3 deletion leads to the overactivation of nonclassical NF-*κ*B signals, whereas the overexpression of TRAF3 inhibits the nonclassical NF-*κ*B signaling pathway [47, 48].

The mechanistic study of nonclassical NF-*κ*B revealed that TRAF3 could not directly induce the ubiquitination and degradation of NIK. TRAF3, TRAF2, NIK, and cIAP exist in the form of complexes in the cytoplasm. The coimmunoprecipitation experiment indicated that TRAF3 and NIK bind together, while TRAF2 and cIAP bind. TRAF3 and TRAF2 are necessary, and they bind together through the TRAF domain and act as a bridge, allowing four proteins to form a complex. cIAP and NIK are close to each other in unstimulated cells due to the junction of TRAF3 and TRAF2, which directly induces K48 ubiquitination and degradation of NIK. Inhibition of cIAP can result in NIK accumulation and NF-*κ*B activation. It has been suggested that cIAP is a ligase that directly induces NIK ubiquitination. More importantly, cIAP-dependent NIK degradation requires TRAF2 expression, while TRAF2-dependent cIAP activity is necessary for causing NIK degradation. TRAF3, cIAP, and TRAF2 were recruited near the cell membrane when LT*β*R, CD40, and BAFFR on the cell surface were stimulated. TRAF2 mediates cIAP K63 ubiquitination, which increases the E3 ubiquitin ligase activity of cIAP, which can then mediate TRAF3 K48 ubiquitination and degradation. Furthermore, it can promote TRAF2 degradation, thereby promoting the accumulation of NIK [49].

These findings suggest that TRAF3 is a platform for mediating the binding of E3 ubiquitin ligase cIAP to substrate NIK, which regulates intracellular NIK levels and, thus, nonclassical NF-*κ*B signals. When the ligand binds to the receptor, TRAF2-dependent cIAP directs the substrate specifically to TRAF3, resulting in TRAF3 degradation, destruction of a link between TRAF3 and NIK, and finally, activation of nonclassical NF-*κ*B signals [50–52].

## 3. Regulatory Mechanism of Modification and Activation of TRAF3

TRAF3 is one of the most versatile members of the TRAF family, and its activation and degradation are mainly regulated by ubiquitin modification. In addition, a complete functional complex is required for TRAF3 to perform its function.

### 3.1. Ubiquitin and Degradation of TRAF3

Ubiquitin modification is an effective protein posttranslational modification in the body. Ubiquitin modification, like other modifications, including protein phosphorylation and methylation, is widely involved in the body's physiological activities. The polyubiquitin chain is mainly formed by N-terminal methionine residue (M1) and seven different lysine residues on ubiquitin, including K6, K11, K27, K29, K33, K48, and K63, respectively. The ubiquitin chains at various sites play different roles. K48-linked ubiquitination and K63-linked ubiquitination are the two main types of ubiquitination. K48 ubiquitin modification mainly mediates the degradation of protein substrate into proteasome, while K63 ubiquitin modification mediates cell signal transduction and plays a regulatory role in stress response and DNA repair [53, 54]. Other ubiquitin modification functions should be investigated further [55].

TRAF3 can produce both K48-linked and K63-linked ubiquitination. It has been identified that K63-linked ubiquitination can occur at K369 and K513 of TRAF3 [56], while K48-linked ubiquitination can occur at K107 and K156 [42]. The K63-linked ubiquitination of TRAF3 is required for its positive regulation, regulating type I interferon production. In contrast, the K48-linked ubiquitination of TRAF3 is essential for terminating its negative regulatory function in classical and nonclassical NF-*κ*B and MAPK signals. It can also inhibit type I interferon production. K48-linked ubiquitination can degrade TRAF3 into the proteasome. In addition, it has been reported that TRAF3 can be degraded into autophagosomes through the receptor protein NDP52 [57]. TRAF3, like TRAF6, functions as an E3 ubiquitin ligase due to its RING structure, with self-ubiquitination of K63 mediated by the RING structure being the key to TRAF3 activation. This process necessitates the participation of E2 ubiquitin ligase UBE2D3 and UBC13. TRAF3 ubiquitination through K63 provides a platform for assembling and activating the NEMO-TBK1/IKK*ε* complex [58]. Ubiquitination and deubiquitination of TRAF3 are critical to regulating interferon production and resisting viral infection [59–61].

PTPN22, HECTD3, TRIM24, cIAP1, and cIAP2 mediate the K63-associated ubiquitination of TRAF3, promoting type I interferon production [62–64]. In contrast, deubiquitinating enzymes such as DUBA, OTUB1, UCHL1, HSCARG, MYSM1, and LGP2 remove the K63-associated ubiquitination of TRAF3 that negatively regulates type I interferon synthesis. PTPN22 can directly bind to TRAF3 and mediate its K63-linked ubiquitination. In contrast, the disease-related mutant PTPN22W cannot mediate the ubiquitination of TRAF3 and cannot upregulate type I interferon, promoting type I interferon-dependent arthritis [62]. HECTD3, an E3 ubiquitin ligase, can mediate K63-linked ubiquitination at K138 of TRAF3, strengthening the TRAF3-TBK1 association and promoting type I interferon production [63]. Vesicular stomatitis virus (VSV) infection causes abundant TRIM24 translocation to mitochondria, which binds to TRAF3 and directly mediates K63-linked TRAF3 ubiquitination at K429/K436. This TRAF3 modification permits it to associate with MAVS and TBK1, which activates downstream antiviral signaling [64]. The apoptosis inhibitors cIAP1 and cIAP2 induce K63-linked ubiquitination of TRAF3 and promote virus-triggered NF-*κ*B and IRF3 activation [47]. Deubiquitin enzyme can remove the K63-linked ubiquitination of TRAF3, destroying the binding of TRAF3 to TBK1 [65–67].

HSCARG can recruit OYUB1, thereby mediating the deubiquitination of TRAF3 with OYUB1. MYSM1 is a histone H2A deubiquitination enzyme [68], and its SWIRM and MPN domains directly bind to TRAF3 and TRAF6, respectively. The binding removes K63-linked ubiquitination and inactivation of TRAF3 and TRAF6, inhibiting the production of inflammatory factors and interferon [69, 70]. LGP2, as a member of the RLR receptor, inhibits the TRAF ubiquitination by binding to the C-terminus of TRAF2/3/5/6, which ultimately inhibits the type I interferon production [71, 72].

In addition to cIAPs, other ubiquitin ligases such as Triad3A, ERa, and WBR82 can mediate K48-associated ubiquitination and degradation of TRAF3 during VSV or Sendai virus (SeV) infection [73–75]. Triad3A can suppress the signaling pathway mediated by RIG, which can degrade TRAF3 to the proteasome by mediating the K48-linked ubiquitination of TRAF3. Triad3A overexpression under double-stranded RNA virus infection reduced the level of TRAF3 in a dose-dependent manner. The amino acid sites Y440 and Q442 of TRAF3 are important for TRAF3, MAVS, and TRAF3 and Triad3A binding. The amino acid sites Y440 and Q442 of TRAF3 could not bind to Triad3A after mutation.

Similarly, the TIM sequence of Triad3A is unable to bind with TRAF3 after mutation. Therefore, Triad3A downregulates the signal transduction of RIG receptors through TRAF3 targeting [73]. ERa, a nuclear receptor family member, can suppress the antiviral immunity induced by the RLR signaling pathway. VSV stimulation can upregulate ERa expression in macrophages. In the case of ligand-independent, VSV infection promotes ERa serine phosphorylation at the 167^th^ position.

Further investigation indicates that ERa inhibits the VSV-induced activation of IRF3 by regulating the K48-linked ubiquitination degradation of TRAF3. Consistent with findings, ERa also inhibits VSV-induced IFN*β* production in macrophages in a ligand-independent manner [74]. Moreover, the TIM sequence of deubiquitinase USP25, 62-PPQEE-66, and 797-PPETDY-802 can bind to TRAF protein and remove the K48 polyubiquitin chain of TRAF3 and TRAF6, improving TRAF3/6 stability [76].

TRAF3 has been reported to undergo K33-linked ubiquitination. The ubiquitination of lysine at position 168 on TRAF3 is mediated by Ral Guanine Nucleotide Dissociation Stimulator (RalGDS). Previous studies demonstrated that bladder epithelial cells quickly eliminate intracellular uremic *E. coli* through the powerful immune defense mechanism of the body to excrete bacteria. TRAF3 binds with RalGDS to ubiquitinate K33 and promotes bacterial excretion through the RalB GTP enzyme-activated cyst complex by activating the TLR4 signal transduction pathway [77].

### 3.2. Phosphorylation of TRAF3

There are few reports on posttranslational modifications of TRAF3 except ubiquitin and deubiquitination. It was reported that serine-threonine kinase Ck1*ε* could bind to TRAF3 and phosphorylate Ser349. The TRAF3 phosphorylation promotes the K63 ubiquitination and the recruitment of TBK1. Ck1*ε*-knockout mice are more susceptible to viral infection. Therefore, Ck1*ε*-mediated TRAF3 phosphorylation establishes an association between TRAF3 ubiquitin and phosphorylation [78].

### 3.3. TRAF3 Complex

The formation of a functional TRAF3 complex is important for TRAF3 activation. Linear ubiquitin ligase LUBAC can reduce the type I interferon synthesis by linear ubiquitination of NEMO. Linear ubiquitinated NEMO can bind to TRAF3, destroy the MAVS and TRAF3 complex, inhibit type I interferon production, and activate the NF-*κ*B signaling pathway. IKK*ε* reduces the binding and stability of MAVS and TRAF3 by facilitating the K63 ubiquitination at position K500 of MAVS, thereby negatively regulating type I interferon [79]. Furthermore, the PLpro-TM of the severe acute respiratory syndrome (SARS) virus can bind to TRAF3, TBK1, IKK*ε*, STING, and IRF3; destroy the STING-TRAF3-TBK1 complex; and reduce the K63 ubiquitination of RIG-I, STING, TRAF3, TBK1, and IRF3 [80, 81].

## 4. TRAF3 and Preclinical Study

### 4.1. Multiple Myeloma

TRAF3 gene deletion or loss of function was identified in tumor cells from patients with multiple myeloma, preventing the binding of TRAF3 and NIK. In addition, a TRAF3 point mutation R118W was also determined that reduces the stability of TRAF3 protein and affects the TRAF3 function. This mutation causes the NIK accumulation and continuous activation of NF-*κ*B, which promotes tumor cell survival. Interestingly, some multiple myelomas have small deletions of NIK, which prevent NIK from binding to TRAF3 and increase its stability consistent with the findings of TRAF3 mutation [82, 83]. In the TRAF3 knockout mice, there was an obvious accumulation of B lymphocytes in secondary lymphoid organs, an increase in serum IgE and autoantibodies, immune complex deposition in the kidney, and B cell infiltration into multiple organs. These mice were predisposed to developing B cell malignant tumors later in life, and aberrant B cell survival was linked to a higher likelihood of developing subsequent mutagenesis events [82]. It has been reported that TRAF3 can exist as a nuclear protein. TRAF3-deficient B cells can survive longer than TRAF3-deficient T cells. Comparing the two types of cells revealed that the cyclic AMP (cAMP) response element-binding (CREB) transcriptional complex increased in TRAF3-deficient B cells. TRAF3 can bind to CREB and its binding proteins in the nucleus, promote CREB ubiquitination and degradation, and inhibit the CREB reporter gene transcriptional activity by recruiting TRAF2-cIAP into the nucleus. TRAF3^−/−^ B cells' survival rate is associated with CREB. In the absence of nuclear TRAF3, the survival-promoting CREB promotes the expression of mRNA and differentiation protein 1 in myeloid leukemia cells [84, 85].

### 4.2. Osteoporosis

Pharmacologic stabilization of TRAF3 during aging could treat/prevent age-related osteoporosis by inhibiting bone destruction and promoting bone formation [86–88].TRAF3 limits bone destruction by inhibiting RANKL-induced NF-*κ*B signaling in osteoclast precursors [89, 90]. Mice with TRAF3 deleted in mesenchymal progenitor cells (MPCs) develop early-onset osteoporosis due to reduced bone formation and enhanced bone destruction. TRAF3 prevents *β*-catenin degradation in MPCs and maintains osteoblast formation of young mice. However, TRAF3 protein levels decrease in murine and human bone samples during aging when TGF*β*1 is released from resorbing bone. TGF*β*1 induces degradation of TRAF3 in murine MPCs and inhibits osteoblast formation through GSK-3*β*-mediated degradation of *β*-catenin. Thus, TRAF3 positively regulates MPC differentiation into osteoblasts [91].

### 4.3. Systemic Lupus Erythematosus

Lupus nephritis (LN) occurs with inflammatory lesion in patients suffering from systemic lupus erythematosus (SLE) [92]. TRAF3 is an important mediator in inflammation. In the LN mouse model, TRAF3 knockdown enhanced the production of IL-10 and reduced the production of proinflammatory cytokines, immunoglobulin, and the expression ofTRAF2, NF-*κ*Bp52, IKK*α*, and ICAM1. TRAF3 plays a role in LN by regulating Th17 cell and Treg cell balance and the NF-*κ*B signaling pathway in mice [93–95].

### 4.4. Non-Hodgkin B Cell Lymphoma

TRAF3 is a master regulator of B cell homeostasis and function. Previous studies showed that TRAF3 overexpression renders B cells hyperreactive to antigens and TLR agonists, while TRAF3 deficiency has been implicated in developing a variety of B cell neoplasms. The latest report showed that transgenic mice overexpressing TRAF3 in B cells develop with high-incidence severe lymphadenopathy, splenomegaly, and lymphoid infiltrations into tissues and organs, which is the result of the growth of monoclonal and oligoclonal B cell neoplasms. These results indicate that TRAF3 may induce mature B cell neoplasms in transgenic mice by promoting exacerbated B cell responses to certain antigens [96].

## 5. TRAF3 and Clinical Study

### 5.1. Herpes Simplex Encephalitis

The R118W mutation of TRAF3 is also found in herpes simplex encephalitis (HSE) patients. This mutation reduces the stability of TRAF3 and the wild-type TRAF3 expression. Therefore, normal functional TRAF3 expression is significantly reduced. The antiviral immune functions of TRAF3-deficient mice decreased as the expression of type I interferon induced by TLR and RIG-I signals decreased significantly. HSE patients had reduced expression of TLR3-TRIF-TRAF3-dependent antiviral response type I interferon, which is consistent with their susceptibility to herpes simplex virus infection [97–100].

### 5.2. Influenza A Virus Infection

TRAF3 could positively regulate innate antiviral response. Overexpression of TRAF3 significantly enhanced virus-induced IRF3 activation, IFN-*β* production, and antiviral response, while TRAF3 knockdown promoted influenza A virus replication. Moreover, inhibiting ubiquitinated degradation of TRAF3 was associated with the anti-influenza effect, thereby facilitating antiviral immunity upon influenza A virus infection. TRAF3 is vital in host defense against influenza A virus infection by the type-I IFN signaling pathway. The findings provide insights into developing drugs to prevent TRAF3 degradation, which could be a novel therapeutic approach for treating influenza A virus infection [101–105].

### 5.3. Celiac Disease

Celiac disease (CD) is an immune-mediated disorder triggered by dietary gluten intake in some genetically predisposed individuals. Regarding clinical studies, differences in the TLR expression and related innate cell activation between active CD patients from one side and controls and treated CD patients from the other side have been described [106]. Even though no specific TLR gene polymorphisms have been associated with CD risk, altered TLR expression (especially for TLR2 and TLR4) has been variably reported in the duodenal mucosal and/or peripheral blood leukocytes from CD patients. Recently, Ghasiyari et al. published a large case-control study (120 CD patients and 120 controls) where mRNA expression of several TLRs (TLR2, TLR4, TLR7, and TLR9) was assessed in the intestinal mucosa for 20 randomly selected samples only. Regarding duodenal mucosa specimens, TLR2 and TLR4 mRNA expression was increased in CD patients compared to controls, whereas TLR9 mRNA expression was significantly decreased in CD patients; no significant difference in the expression of TLR7 mRNA was observed between the study groups [107]. On the other hand, celiac diseases are related to HLA-related genetic factors. Selective IgA deficiency (SIgAD) is the most frequent primary immune defect. The genetic background of SIgAD is complex, and three HLA haplotypes resulted in being more frequently associated with it; in detail, two of them include HLA-DQB1∗02 allelic variants, which are essential predisposing factors in developing CD [108]. However, it is not clear how TRAF3 is critical for CD so far.

### 5.4. Primary Immunodeficiency

Complete defects in two main TLR-dependent pathways have been described so far. One defect leads to greater susceptibility to pyogenic bacteria (MyD88-IRAK4 deficiency), and the other results in greater susceptibility to herpesviruses (TLR3-UNC93B1-TRIF-TRAF3 deficiency). It has been reported that patients with an autosomal dominant mutation of TRAF3 are characterized by recurrent herpesvirus encephalitis. Interestingly, this immunodeficiency syndrome leads to greater susceptibility only to herpesvirus encephalitis and no diseases caused by other pathogens. The functional defect in patients with such deficiency in TLR3-UNC93B1-TRIF-TRAF3 is probably the result of less capacity to release type I interferons [109].

### 5.5. Guillain-Barré Syndrome

Guillain-Barré syndrome (GBS) is the commonest postinfectious polyradiculopathy. Dysregulation of TLR molecules exacerbates immune-inflammatory responses, and the genetic variations within TLR pathway-related genes contribute to the differential risk of infection. It has been shown that genotypes of two polymorphic variants, Del/Del of rs111200466 insertion and deletion polymorphism of TLR2 gene and TT of rs3775290 single nucleotide polymorphism (SNP) of TLR3 gene, had significantly higher frequencies among GBS patients, while the frequencies of TT genotype of rs3804099 SNP of TLR2 gene and TT genotype of rs11536891 SNP of TLR4 gene were significantly higher in healthy subjects. The genes encoding TLRs and TLR signaling pathway-related molecules could serve as crucial genetic markers of susceptibility and severity of GBS. But the TRAF3 gene was not found to be associated with GBS risk by analyzing the linkage disequilibrium [110].

## 6. Conclusions

TRAF3 is the most unique and versatile member of the TRAF family. In recent years, the functions and regulatory mechanisms of TRAF3 in its dependent signaling pathways have been thoroughly elucidated, providing a new target for the clinical prevention and treatment of some tumors, viral infections, and other diseases. In this review, we summarize and discuss the evidence from preclinical studies and clinical studies as regards the potential role of TRAF3 in disease, as schematically summarized in Table 1. Targeted treatment of TRAF3 or TLR is beneficial to treating autoimmune diseases or conditions. However, our current understanding of TRAF3 needs to be completed, and many questions still need to be answered, such as how to recruit proteins that bind to TRAF3 to perform different functions in different signal complexes. What proteins are the ubiquitin substrate of K63 mediated by TRAF3? How does TRAF3 play a role in other autoimmune diseases? These problems must be studied in the future.

## Figures and Tables

**Table 1:** TRAF3 in immune diseases.

Disease	Mechanism	References
*Preclinical study*		
Multiple myeloma	The whole gene or loss of function of TRAF3 hindered the binding of TRAF3 and NIK	[82]
Osteoporosis	TRAF3 limits bone destruction by inhibiting RANKL-induced NF-*κ*B signaling in osteoclast precursors	[90]
Systemic lupus erythematosus	TRAF3 regulates Th17 cell and Treg cell balance and the NF-*κ*B signaling pathway	[95]
Non-Hodgkin B cell lymphoma	Overexpression of TRAF3 in B cells	[96]
*Clinical study*		
Herpes simplex encephalitis	The R118W mutation of TRAF3	[97]
Influenza A virus infection	Overexpression of TRAF3 enhanced virus-induced IRF3 activation, IFN-*β* production, and antiviral response	[102]
Celiac disease	TLR2 and TLR4 mRNA expression was increased in CD patients	[106]
Primary immunodeficiency	Autosomal dominant mutation of TRAF3	[109]
Guillain-Barré syndrome	The genetic variations within TLR2 and TLR3	[110]

## Data Availability

The data used to support the findings of this study are included within the article.
